# First Detection in West Africa of a Mutation That May Contribute to Artemisinin Resistance *Plasmodium falciparum*

**DOI:** 10.3389/fgene.2021.701750

**Published:** 2021-10-08

**Authors:** Hui Zhao, Liang Pi, Luyi Zhao, Yucheng Qin, Weilin Zeng, Zheng Xiang, Qi Yang, Maohua Pan, Xinxin Li, Chunyan Zou, Xi Chen, Wei Zhao, Yuxin Lu, Yanrui Wu, Mengxi Duan, Xun Wang, Xiaosong Li, Dominique Mazier, Yaming Huang, Zhaoqing Yang

**Affiliations:** ^1^Department of Pathogen Biology and Immunology, Kunming Medical University, Kunming, China; ^2^Shanglin County People's Hospital, Guangxi, China; ^3^Guangxi Zhuang Autonomous Region People's Hospital, Nanning, China; ^4^Department of Cell Biology & Genetics, Kunming Medical University, Kunming, China; ^5^Sorbonne Université, INSERM, CNRS, Centre d'Immunologie et des Maladies Infectieuses, CIMI, Paris, France; ^6^Guangxi Zhuang Autonomous Region Center for Disease Prevention and Control, Nanning, China

**Keywords:** *Plasmodium falciparum*, antimalarial drug, polymorphism, drug resistance genes Pfkelch13, Pfcrt, Pfmdr1

## Abstract

**Background:** The spread of drug resistance has seriously impacted the effective treatment of infection with the malaria parasite, *Plasmodium falciparum*. Continuous monitoring of molecular marker polymorphisms associated with drug resistance in parasites is essential for malaria control and elimination efforts. Our study describes mutations observed in the resistance genes *Pfkelch13, Pfcrt*, and *Pfmdr1* in imported malaria and identifies additional potential drug resistance-associated molecular markers.

**Methods:** Chinese patients infected in Africa with *P. falciparum* were treated with intravenous (IV) injections of artesunate 240–360 mg for 3–5 days while hospitalized and treated with oral dihydroartemisinin-piperaquine (DHP) for 3 days after hospital discharge. Blood samples were collected and PCR sequencing performed on genes *Pfkelch13, Pfcrt*, and *Pfmdr1* from all isolates.

**Results:** We analyzed a total of 225 patients from Guangxi, China with *P. falciparum* malaria acquired in Africa between 2016 and 2018. All patients were cured completely after treatment. The F446I mutation of the *Pfkelch13* gene was detected for the first time from samples of West African *P. falciparum*, with a frequency of 1.0%. Five haplotypes of *Pfcrt* that encode residues 72–76 were found, with the wild-type CVMNK sequence predominating (80.8% of samples), suggesting that the parasites might be chloroquine sensitive. For *Pfmdr1*, N86**Y** (13.1%) and Y184**F** (58.8%) were the most prevalent, suggesting that artemether-lumefantrine may not, in general, be a suitable treatment for the group.

**Conclusions:** For the first time, this study detected the F446I mutation of the *Pfkelch13* gene from Africa parasites that lacked clinical evidence of resistance. This study provides the latest data for molecular marker surveillance related to antimalarial drug resistance genes *Pfkelch13, Pfcrt*, and *Pfmdr1* imported from Africa, in Guangxi, China from Chinese migrate workers.

**Clinical Trial Registration:** ChiCTROPC17013106.

## Introduction

Malaria is a serious public health problem in tropical and subtropical areas of the world. There were ~229 million cases and 409,000 malaria-related deaths worldwide in 2019, with Africa shouldering over 94% of the global malaria burden (WHO, 2020). While five species of *Plasmodium* can cause human disease, *Plasmodium falciparum* is most notorious due to high levels of mortality and morbidity associated with infection. The recent decline in malaria prevalence is mainly due to the application of efficacious antimalarial drugs, early diagnosis by RDT, and distribution of long-lasting insecticide-treated nets (LLTINs) in malaria- endemic areas. However, the emergence of drug resistance remains a persistent obstacle to control and eliminate malaria. Here we sought to better understand the geography of resistance alleles in hopes of guiding clinical decisions and support malaria surveillance and elimination programs.

Imported malaria is increasing in China owing to increasing global economic integration. Disease among migrant laborers attributable to *P. falciparum* has increased rapidly in recent years (Feng et al., 2014; Liu et al., 2014). From 2011 to 2016, 19,154 imported cases were reported in mainland China, most of which came from Africa (72%) (Lai et al., 2019). In the past, China suffered seriously from endemic malaria, but the occurrence of malaria has been reduced; 2017 marked the first time that no locally acquired cases were reported (Feng et al., 2018). The World Health Organization (WHO) deemed China to have achieved malaria elimination in 2021. Thus, imported disease has become the main challenge remaining to eliminate malaria from China. It is particularly important to continue to monitor malaria imported into China through molecular monitoring. Chinese migrant workers lack prior immunity; thus, most self-administer antimalarial drugs when working in endemic regions, often without medical guidance; such practices may induce drug resistance. Understanding the emergence of drug resistance therefore requires attention to the behaviors and consequences of such migrant workers (Yang et al., 2017; Wang et al., 2018, 2020; Yao et al., 2018; Li et al., 2019; She et al., 2020; Zhao et al., 2020; Shi et al., 2021).

Antimalarial drugs are principal tools for malaria control. In Africa in the 1990s, chloroquine (CQ) was used to treat malaria and saved tens of millions of lives (Trape, 2001). Chloroquine was once highly efficacious against uncomplicated *P. falciparum* infection. Widespread use engendered resistance (CQR) (Young et al., 1963; Fogh et al., 1979; Wellems and Plowe, 2001); the WHO then recommended artemisinin-based combination therapies (ACTs) as the first-line antimalarial treatment of choice. According to a WHO report, Côte d'Ivoire and Gabon used artesunate-amodiaquine as a first-line treatment for *P. falciparum* in 2003 and artesunate-amodiaquine as a first-line treatment for *P. falciparum* in Cameroon and Liberia in 2004. Mozambique in 2004 used artemether-lumefantrine as a first-line treatment for *P. falciparum*. In Sierra Leone and Ghana, artemether-lumefantrine and artesunate-amodiaquine were used as first-line treatments for *P. falciparum* in 2004, and artemether-lumefantrine and artesunate-amodiaquine were used as first-line treatments for *P. falciparum* in Mali in 2007 (WHO, 2015). Artemether-lumefantrine is used widely in African countries.

Artemisinin resistance (ART-R) has emerged in Africa (Lu et al., 2017a), and its appearance is a major threat to malaria control efforts including those efforts aimed at treating imported malaria. Mutations in the Kelch propeller gene (*Pfkelch13*) have, elsewhere, been linked to reduced efficacy of artemisinin. Particular mutations in the *Pfkelch13* gene discovered to play an important role in ART-R include Y439H, F446I, M476I, R539T, I543T, and C580Y increasing ring-stage survival (RSA) (Straimer et al., 2015; Mita et al., 2016; Zhang et al., 2017; Wang et al., 2018). Mutations that have been detected in Africa samples include T149S, K189T/N, P441S, S459T, D464E, C469F, T474I, A481T, K503E, R539T, R561H, P574L, A578S, C580Y, V589I, V603E, E612K, Q613E, R622I, V650F, G665S, A675V, V692L, and N694K, but not F446I (Conrad et al., 2014a; Torrentino-Madamet et al., 2014; Tacoli et al., 2016; Ikeda et al., 2018; Wang et al., 2018, 2020; Yao et al., 2018; Li et al., 2019; Ocan et al., 2019; She et al., 2020).

Alleles of the *P. falciparum* chloroquine resistance transporter gene (*Pfcrt*) have been identified as molecular markers of CQR (Warhurst, 2001; Ariey et al., 2014). These include mutations in amino acids 72–76 (Awasthi and Das, 2013) with K76T being the most common change found in CQR parasites. The *P. falciparum* multidrug resistance protein-1 (*Pfmdr1*) has also been associated with resistance to multiple antimalarial drugs. Mutations at residues 86, 184, 1034, 1042, and 1246 have been shown to be associated with resistance to CQ, amodiaquine (AQ), quinine (QN), mefloquine (MQ), halofantrine (HF), and artemisinin (ART) (Duraisingh et al., 1997; Sidhu et al., 2005; Humphreys et al., 2007; Lekostaj et al., 2008; Tinto et al., 2008; Folarin et al., 2011).

In Africa, artemisinin resistance has emerged, and its appearance threatens current efforts to control malaria (Lu et al., 2017b). Antimalarial drug use among Chinese laborers may contribute to drug pressures. Therefore, monitoring the molecular markers of drug resistance in imported malaria cases complements studies in endemic African populations, enriching the picture of polymorphisms that may contribute to drug resistance. Here, we describe the distribution of *Pfkelch13, Pfcrt*, and *Pfmdr1* from *P. falciparum* isolates imported from Africa to Guangxi, South China from 2016 to 2018.

## Materials and Methods

### Sample Collection and Treatment

All samples were collected between 2016 and 2018 from Chinese migrant workers that had returned from Africa and who were diagnosed at the Guangxi Shanglin Hospital with uncomplicated *falciparum* malaria. The diagnosis was based on the microscopy of Giemsa-stained blood smears. Venous blood samples (2–5 ml) were collected before treatment. Prospective cases with complex travel histories were excluded from the study (Zhao et al., 2020). All uncomplicated patients were treated with intravenous (IV) injections of artesunate, on day 0 with 120 mg, and days 1, 2, 3, and 4 with 60 mg on each subsequent day during hospitalization. Upon resolution of symptoms, the patients were discharged, and 3 days of oral treatment with dihydroartemisinin and piperaquine phosphate tablets (dihydroartemisinin-piperaquine, DHP, dihydroartemisinin 40 mg, and piperaquine phosphate 320 mg for each tablet) was prescribed, on day 1, day 2, and day 3 with 4 pills, 2 pills, and 2 pills, respectively.

This study was approved by the Ethics Review Committees of the Institutional Review Board (IRB) of Shanglin Hospital. All participants supplied written informed consent. This study was registered on the Chinese Clinical Trial Registry with Registration Number ChiCTROPC17013106.

### DNA Extraction and PCR

Parasite genomic DNA was isolated from 2 to 5 ml of venous blood using the High Pure PCR Template Preparation Kit (Roche Biotech Co., Ltd., Germany). The template DNA was amplified by Nest-PCR-sequence of the *Pfkelch13* gene (Gene ID: PF3D7_1343700; Wang et al., 2015), a 145-bp fragment of the *Pfcrt* gene (Gene ID: PF3D7_0709000; Zhou et al., 2016), and two fragments (N1: 526 bp and N2: 799 bp) of the *Pfmdr1* gene (Gene ID: PF3D7_0523000; Li et al., 2015), including the entire sequence of the *Pfkelch13* gene, *Pfcrt* mutations at codons 72–76, and *Pfmdr1* mutations at codons 86, 130, 184, 1034, 1042, 1109, and 1246. PCR products were purified, sequenced by the Sangon Biotech Co Ltd (Kunming, China), and analyzed using DNAstar (DNASTAR Inc., Madison, WI, USA).

### Statistical Analyses

Experimental data were analyzed by SPSS 19.0 (SPSS Inc. Chicago, IL, USA). The variation tendency for haplotypes and the frequency mutations of *Pfkelch13, Pfcrt*, and *Pfmdr1* over the study period was evaluated by chi-square test; the trend among the groups from patients from different regions was assessed by chi-square test with *p* < 0.05 being considered significant.

## Results

### Epidemiologic Date of Cases and Clinical Outcome

A total of 225 uncomplicated *P. falciparum* cases that returned from Africa to Guangxi Province between 2016 and 2018 were analyzed in this study. None of the patients had traveled to any other areas prior to, or after, spending time in Africa (Zhao et al., 2020). Among the infected individuals, most (77.8%, 175/225) had spent time in West Africa, with 20.4% (46/225) and 1.8% (4/225) having been in Central and South Africa, respectively (Supplementary Table 1). The majority of the patients who returned from West Africa had been in either Kumasi or Asankragua in Ghana. The patients' clinical information is included in Supplementary Table 2. Fever was resolved in 80% of the patients within 2 days of treatment. Patients were discharged on days 3, 4, and 5 (15, 70, and 15%, respectively). Six months after discharge, the patients reported no additional fever.

### Prevalence of Mutations in *Pfkelch13, Pfcrt*, and *Pfmdr1* Genes

**Figure 1:**
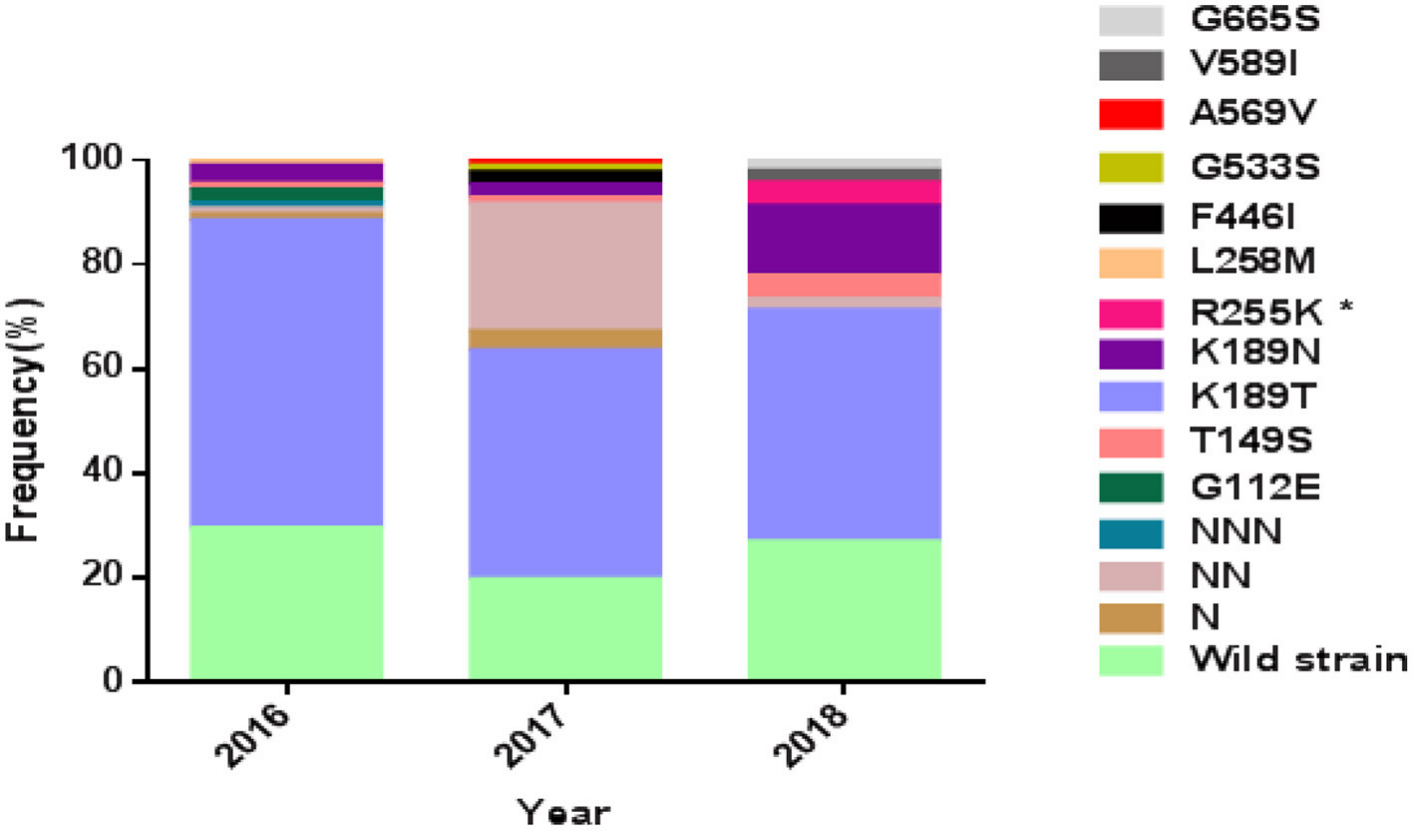
Prevalence of PfK13 mutations from studied isolates. *The difference is statistically significant between 2016 and 2018.

The complete *Pfkelch13* gene was successfully amplified and sequenced from 92.4% (208/225) of the samples. The sequences were deposited in GenBank (MK877250- MK877457). The distribution of *Pfkelch13* mutations is shown in Figure 1 and Tables 1, 2.

**Table 1:** Prevalence rate of wild and mutant *Pfkelch13* gene from studied isolates.

**Mutants**	**No. (%) of samples from Western Africa**	**No. (%) of samples from Non-Western Africa**	**No. (%) total**	***p*-value**
Wild strain	32 (20)	21 (43.7)	53 (25.5)	<0.001^*^
**N** insertion	3 (1.9)	1 (2.1)	4 (1.9)	0.93
**NN** insertions	21 (13.1)	1 (2.1)	22 (10.6)	0.03^*^
**NNN** insertions	1 (2.1)	0 (0.0)	1 (0.5)	0.58
G112**E**	2 (1.2)	0 (0.0)	2 (1.0)	0.44
T149**S**	2 (1.2)	2 (4.2)	4 (1.9)	0.20
K189**T**/**N**	93 (58.1)	24 (50)	117 (56.2)	0.32
R255**K**	1 (0.6)	1 (2.1)	2 (1.0)	0.36
L258**M**	0 (0.0)	1 (2.1)	1 (0.5)	0.07
F446**I**	2 (1.2)	0 (0.0)	2 (1.0)	0.44
G533**S**	1 (0.6)	0 (0.0)	1 (0.5)	0.58
A569**V**	1 (0.6)	0 (0.0)	1 (0.5)	0.58
V589**I**	1 (0.6)	0 (0.0)	1 (0.5)	0.58
G665**S**	1 (0.6)	0 (0.0)	1 (0.5)	0.58

* *The difference is statistically significant*.

**Table 2:** *Pfkelch13* mutations from studied isolates.

**Mutation**	**Type**	**Source countries**
**N** insertion	NS	Ghana (*N* = 3) Cameroon (*N* = 1)
**NN** insertions	NS	Ghana (*N* = 20) Cameroon (*N* = 1) Liberia (*N* = 1)
**NNN** insertions	NS	Ghana (*N* = 1)
L119L^*^	S	Ghana (*N* = 3) Liberia (*N* = 1)
T149**S**	NS	Ghana (*N* = 2) Cameroon (*N* = 1) Congo (*N* = 1)
K189**T**	NS	Ghana (*N* = 81) Cameroon (*N* = 16) Congo (*N* = 3) Central African Republic (*N* = 2) Mozambique (*N* = 1)
		Sierra Leone (*N* = 1) Mali (*N* = 1) Gabon (*N* = 1)
K189**N**	NS	Ghana (*N* = 8) Mozambique (*N* = 1) Liberia (*N* = 2)
R255**K**	NS	Liberia (*N* = 1) Cameroon (*N* = 1)
L258M^*^	NS	Central African Republic (*N* = 1)
Y288Y^*^	S	Ghana (*N* = 1)
F446**I**	NS	Ghana (*N* = 2)
T456C^*^	S	Ghana (*N* = 1)
C469C	S	Ghana (*N* = 2)
Y493Y^*^	S	Ghana (*N* = 1)
G496G^*^	S	Ghana (*N* = 2)
G533**S**	NS	Ghana (*N* = 1)
A569V^*^	NS	Ghana (*N* = 1)
V589**I**	NS	Ghana (*N* = 1)
A627A	S	Cote d'Ivoire (*N* = 1)
G665S^*^	NS	Ghana (*N* = 1)
A676A^*^	S	Sierra Leone (*N* = 1) Cameroon (*N* = 1)
G690G^*^	S	Ghana (*N* = 1) Mali (*N* = 1)

*S, synonymous mutations; NS, non-synonymous mutations.*

* *Mutation was unreported before*.

The wild-type allele accounted for 25.5% of the samples and was found in higher frequency in samples from patients who had been in areas other than West Africa (*p* < 0.01). A total of 19 single nucleotide polymorphisms (SNPs), including 9 synonymous and 10 non-synonymous changes, were observed in the gene, of which 6 non-synonymous (T149S, K189T/N, R255K, F446I, G533S, and V589I) had been reported previously (Wang et al., 2015; Boussaroque et al., 2016; Yao et al., 2018) while 4 (G112E, L258M, A569V, and G665S) were new (Table 1). One, two, or three asparagine insertions at codon 142 were found in multiple samples (13.00%, 27/208). The non-synonymous mutations at K189T/N or T149S were most prevalent, being found in 50.9, 5.3, and 1.9% of the samples, respectively (Figure 1). In this study, the mutation F446I was observed in two of the isolates from Ghana (Figure 1 and Table 2, GenBank accession numbers MK877371 and MK877381).

From the 225 samples, the *Pfcrt* and *Pfmdr1* genes were successfully sequenced from 193 (85.8%) to 204 (90.7%), respectively. The sequences were deposited in GenBank (MZ572562-MZ572754, MZ572358-MZ572561, MZ577609-MZ577812). The mutations found in *Pfcrt* and *Pfmdr1* are indicated in Tables 3–5. For *Pfcrt*, there were no polymorphisms at position 72 or 73. Mutations leading to changes at positions M74**I** and N75**E** were found in the same abundance, making up 15.5% (30/193) of the total samples analyzed, while mutations impacting residue 76 were most frequent (17.6%; Table 3). *Five* haplotypes within *Pfcrt* residues 72–76 were identified, including CVMNK (wild type), CV**IET**, CV**IE**K, CVMN**T** (mutant types), and CVM/**I** N**/E** K/**T** (mixed types) with prevalence of 80.8% (156/193), 13.5% (26/193), 1.6% (3/193), 3.6% (7/193), and 0.5% (1/193), respectively (Table 4). For *Pfmdr1*, the prevalence of polymorphisms impacting positions 184 and 86 totaled 58.8 and 13.1%, respectively. Other, less frequent mutations included D89**G** (0.5%), F1226**Y** (3.9%), and D1246**Y** (2.4%). No mutations at position 1034 or 1042 were detected (Table 3). Eleven haplotypes with differences at residues 86, 184, and 1246 were found, including wild type (NYD), mutation haplotypes (**Y**YD, N**F**D, **YF**D, and **Y**Y**Y**), and mixed haplotypes (N/**Y** YD, N/**Y F**D, N Y/**F** D, **Y** Y/**F** D, N/**Y** Y/**F** D, and **Y** Y/**F** D/**Y**), accounting for 34.3, 4.4, 41.7, 2.9, 2.0, 0.5, 0.5, 10.7, 0.5, 2.0, and 0.5%, respectively (Table 4). Among the haplotypes from samples from migrants from West and Non-West Africa, the wild type (NYD) was found with similar frequency (*p* > 0.05). The N**F**D and **YF**D haplotypes show a significant difference among the groups (*p* < 0.05). The haplotype frequency of *Pfmdr1* **Y**YD was markedly reduced during the time period of the study, accounting for 8.3% of the diversity in 2016 but undetected in 2018 (*p* < 0.05; Table 5). The distribution of all *Pfmdr1* haplotypes is shown in Table 5.

**Table 3:** Polymorphisms of *Pfcrt* and *Pfmdr1* in studied isolates.

**Gene**	**Codons position**	**No. of isolates**	**Prevalence of mutation (%)**
*Pfcrt* (*N* = 193)	M74**I**	30	15.5
	N75**E**	30	15.5
	K76**T**	34	17.6
*Pfmdr1* (*N* = 204)	N86**Y**	27	13.1
	D89G^*^	1	0.5
	Y184**F**	120	58.8
	F1226**Y**	8	3.9
	D1246**Y**	5	2.4

* *Mutation was unreported before*.

**Table 4:** Distribution of *Pfcrt* and *Pfmdr1* haplotypes from studied isolates.

**Gene**	**Haplotype**	**No. (%) of samples from West Africa**	**No. (%) of samples from Non-West Africa**	**No. (%) total**	***p*-value**
*Pfcrt*	CVMNK(wild type)	123 (81.5)	33 (78.6)	156 (80.8)	0.674
Codon 72–76	CV**IET**	19 (12.6)	7 (16.7)	25 (13.5)	0.493
	CV**IE**K	2 (1.3)	1 (2.4)	3 (1.6)	0.624
	CVMN**T**	6 (3.9)	1 (2.4)	7 (3.6)	0.625
	CVM/**I** N**/E** K/**T**	1 (0.7)	0 (0.0)	1 (0.5)	0.597
	Total	151	42	193	
*Pfmdr1*	NYD(wild type)	50 (31.2)	20 (45.4)	70 (34.3)	0.079
Codon 86, 184, 1246	**Y**YD	5 (3.1)	4 (9.1)	9 (4.4)	0.088
	N**F**D	78 (48.7)	7 (15.9)	85 (41.7)	<0.0001^*^
	**YF**D	2 (1.2)	4 (9.1)	6 (2.9)	0.006^*^
	**Y**Y**Y**	3 (1.8)	1 (2.3)	4 (2.0)	0.866
	N/**Y** YD	1 (0.6)	0 (0.0)	1 (0.5)	0.326
	N/**Y F**D	1 (0.6)	0 (0.0)	1 (0.5)	0.599
	N Y/**F** D	15 (9.4)	7 (15.9)	22 (10.7)	0.216
	**Y** Y**/F D**	1 (0.6)	0 (0.0)	1 (0.5)	0.599
	N/**Y** Y/**F** D	3 (1.9)	1 (2.3)	4 (2.0)	0.866
	**Y** Y**/F** D**/Y**	1 (0.6)	0 (0.0)	1 (0.5)	0.599
	Total	160	44	204	

* *The difference is statistically significant*.

**Table 5:** Haplotypes of *Pfcrt* and *Pfmdr1* from studied isolates^*a*^.

**Gene**	**Haplotype*^***b***^***	**Year No. (%)**			
		**2016**	**2017**	**2018**	***p*-value**
*Pfcrt*	CVMNK	61 (84.7)	60 (75.0)	35 (85.4)	0.818
	CVIET	11 (15.3)	11 (13.8)	4 (9.8)	0.426
	CVIEK	0 (0.0)	3 (3.7)	0 (0.0)	0.708
	CVMNT	0 (0.0)	6 (7.5)	1 (2.4)	0.274
	CVM/I N/E K/T	0 (0.0)	0 (0.0)	1 (2.4)	0.120
	Total	72	80	41	
*Pfmdr1*	NYD	23 (27.4)	33 (44.6)	14 (30.4)	0.209
	**Y**YD	7 (8.3)	2 (2.7)	0 (0.0)	0.019^c^
	NFD	38 (45.2)	26 (35.1)	21 (45.6)	0.831
	**YF**D	4 (4.8)	1 (1.4)	1 (2.2)	0.315
	**Y**YY	2 (2.4)	2 (2.7)	0 (0.0)	0.414
	N/Y YD	0 (0.0)	1 (1.4)	0 (0.0)	0.810
	N/Y FD	0 (0.0)	0 (0.0)	1 (2.2)	0.126
	N Y/F D	10 (11.9)	5 (6.7)	7 (15.2)	0.749
	**Y** Y/F D	0 (0.0)	1 (1.4)	0 (0.0)	0.810
	N/Y Y/F D	0 (0.0)	3 (4.0)	1 (2.2)	0.209
	**Y** Y/F D/Y	0 (0.0)	0 (0.0)	1 (2.2)	0.125
	Total	84	74	46	

a *The haplotypes were constructed considering codon positions 72–76 of Pfcrt and codon positions 86, 184, and 1246 of Pfmdr1.*

b *Amino acid mutation is in bold type.*

c *The difference is statistically significant*.

## Discussion

In our study, several antimalarial molecular markers (*Pfkelch13, Pfcrt, Pfmdr1*) were used to monitor drug resistance in imported *P. falciparum* cases returning from Africa to Shanglin County of Guangxi Province during 2016–2018. Resistance to artemisinin, a widely used front-line antimalarial drug, is increasing. Mutations in the *Pfkelch13* gene have served as useful molecular markers for ART-R (Ariey et al., 2014). To date, more than 200 non-synonymous mutations in the *Pfkelch13* gene have been reported (MalariaGEN Plasmodium falciparum Community Project, 2016; WHO, 2018), and several validated resistance mutations in the *Pfkelch13* gene have been described that are associated with delayed parasite clearance *in vitro* or *in vivo*, or both. These include F446I, N458Y, M476I, Y493H, R539T, I543T, P553L, R561H, and C580Y (Amaratunga et al., 2014a,b; Ariey et al., 2014; Ashley et al., 2014; Huang et al., 2015; Straimer et al., 2015; Takala-Harrison et al., 2015; WHO, 2018). These changes have been found in distinct geographic regions. For example, F446I, R539T, I543T, P553L, and C580Y have been found in high prevalence in the Greater Mekong Subregion (GMS) (Wang et al., 2015), while the most frequent mutation found along the Thailand–Myanmar border and in Cambodia, Thailand, and Laos is the C580Y variant (Ariey et al., 2014; Menard et al., 2016; Phyo et al., 2016; Imwong et al., 2017). Previous research has shown that the mutations at residues 539 and 580 were also observed in migrant workers returning from Ghana (Feng et al., 2015a), and other mutations were detected from African samples, including T149S, K189T/N, P574L, Q613E, and A675V (Conrad et al., 2014a; Torrentino-Madamet et al., 2014; Tacoli et al., 2016; Ikeda et al., 2018). In this study, two Ghanaian samples with mutations known to convey ART-R phenotypes were found at residue 446 of the *Pfkelch13* gene, marking the first time this genotype has been identified in samples from West Africa. As a validated resistance mutation in the *Pfkelch13*, the mutant F446I is mainly found proximal to the China–Myanmar border and has been associated with high RSA (Zhang et al., 2017; Wang et al., 2018) and delayed parasite clearance (Feng et al., 2015b; Huang et al., 2015; Wang et al., 2018). It has not been detected before in Africa.

The infections of patients treated in this study with IV artesunate followed by oral DHP resolved 3–5 days thereafter, suggesting that ART-R succeeded even against parasites bearing the F446I mutation. The clinical success may relate to the use of IV treatment with artesunate, which is typically reserved for severe cases but which was used for all patients in this study. Treatment modality deserves further consideration as an explanation for clinical success in these instances (Wang et al., 2019). It may also be true that F446I-bearing parasites remained sensitive to artemisinin owing to the presence (or absence) of additional mutations relevant to drug sensitivity. Among all samples, the highest frequency of mutations was K189T/N, T149S, or an insertion of N, NN, and NNN, which is consistent with a previous report from Dakar (Torrentino-Madamet et al., 2014). However, there is no definitive evidence that the mutations at 189 and 149 are related to ART-R (Miotto et al., 2015).

Only three residues (amino acids 74, 75, and 76) were found to be mutated in the *Pfcrt* gene, of which 76 had the highest frequency of change with 17.6% prevalence. No mutations were found at positions 72 and 73, which was consistent with previous studies of *P. falciparum* imported to Shandong Province, China (Xu et al., 2018). Previous work has shown that the *Pfcrt* 76**T** mutation was the main marker for CQR (Fidock et al., 2000), while research conducted in Nigeria demonstrated an association between the 76**T** mutation in the *Pfcrt* gene and reduced susceptibility to artemether *in vitro* (Bustamante et al., 2012). In addition, increased frequency of the 76**T** allele raises concerns about the use of ACTs because it has been found after using artemether-lumefantrine (AL) and dihydroartemisinin-piperaquine (DHAP) in Burkina Faso (Baraka et al., 2015). We also found five haplotypes of the *Pfcrt* gene at positions 72–76, including CVMNK (wild type), CV**IET**, CV**IE**K, CVMN**T** (mutation type), and CVM/**I** N**/E** K/**T** (multiple clonal infection). The prevalence of the *Pfcrt* wild-type haplotype was high (80.8%), which is consistent with a previous report of the prevalence of this allele in Ghana (90%; Osarfo et al., 2018). This result was also similar to a recent report on imported malaria, in which the prevalence of wild-type *Pfcrt* CQ sensitive parasites from different regions of Africa was dominant (Lu et al., 2017c). In this study, CV**IET** (13.5%) was more frequently observed than other mutant haplotypes, and this finding was similar in samples of malaria parasites acquired from either West Africa or Non-West African regions. The prevalence of molecular markers associated with CQ resistance has decreased since changes in treatment have been enacted (Conrad et al., 2014b). For example, in Malawi and Tanzania, *Pfcrt* alleles associated with CQ sensitivity have reemerged after CQ withdrawal (Kublin et al., 2003; Laufer et al., 2006; Mohammed et al., 2013). This suggests that CQ could possibly be reintroduced as an effective antimalarial drug in the future (Duah et al., 2013; Lu et al., 2017c).

Mutations in the *Pfmdr1* gene at residues 86, 184, 1034, 1042, and 1246 have been reported to be associated with resistance to multiple antimalarial drugs. In our study, we found five non-synonymous mutations at positions 86, 89, 184, 1226, and 1246. The main mutation sites were 86 and 184, being found in 13.1 and 58.8% of the samples, respectively. As far as we know, D89**G**, which we detected at a frequency of 0.5%, has not been described before as a resistance allele. Its role in drug resistance remains to be confirmed. The F1226**Y** mutation has previously been detected in imported isolates from Africa (Yao et al., 2018). Together, our data and the literature have revealed that at least 11 haplotypes are possible at positions 86, 184, and 1246 in the *Pfmdr1* gene. The high-frequency N86, 184**F**, and D1246 alleles have been previously reported to be correlated with selection *in vivo* with AL (Vinayak et al., 2010; Baliraine and Rosenthal, 2011; Thomsen et al., 2011, 2013). A mutation N86Y in Pfmdr1 increases sensitivity to lumefatrine and mefroquine (Menard and Dondorp, 2017). In Africa, the treatment of uncomplicated malaria has changed from a CQ-based approach to one based on AL and DHAP (Duah et al., 2013; Gadalla et al., 2013). The difference of N**F**D and **YF**D that we have observed among imported samples from West Africa and Non-West Africa may be related to subtle differences in treatment schemes (and thereby selective pressure) among the countries in Africa.

In conclusion, this study detected the prevalence of polymorphisms in *Pfkelch13, Pfcrt*, and *Pfmdr1* genes in Chinese migrant workers returning to Guangxi Province. Mutations at residue 446 in the *Pfkelch13* gene, which is associated with ART-R(Wang et al., 2018), was detected for the first time in authentic malaria samples from West Africa in our study, but it did not coincide with clinical evidence of ART-R. *Pfcrt* gene polymorphisms were rare, and the wild-type haplotype was more frequently identified, suggesting that CQ could be used as an effective antimalarial drug in the future. The frequencies of *Pfmdr1* N86**Y** and Y184**F** alleles were most abundant, while the D1246**Y** allele was low. These observations suggest that AL may not be an effective choice for treatment.

## Data Availability Statement

The raw data supporting the conclusions of this article will be made available by the authors, without undue reservation.

## Author Contributions

HZ, LP, and LZ: conceptualization, investigation, writing—original draft, and visualization. YQ: validation and investigation. WZe: investigation, methodology, supervision. WZe and MP: validation and data curation. ZX: data curation. YW: visualization and supervision. DM: writing—review and editing. YH: resources: project administration and conceptualization. ZY: conceptualization, methodology, and funding acquisition. QY, XinL, CZ, YL, WZh, MD, XiaL, and XW: resources and validation. All authors contributed to the article and approved the submitted version.

## Funding

This study was supported by the National Science Foundation of China (31860604 and U1802286), major science and technology projects of Yunnan Province (2018ZF0081), and the International Science and Technology Cooperation–Yunnan International Science and Technology Cooperation Base (202003AE140004). YL was funded by grants of the Science and Technology Bureau Programs for Science and Technology Development of Nanning (ZC20153012). YQ was funded by grants of Guangxi Zhuang Autonomous Region Health Commission of Scientific Research Project (Z20190892). CZ was funded by grants of the Youth Fund Project of People's Hospital of Guangxi Zhuang Autonomous Region, China (No. QN2017-10). WZe was under the support of the Education Department Fund of Yunnan Province (2019J1184). XC and ZX were under sponsoring from the Yunnan Applied Basic Research Projects–Union Foundation [2018FE001-190 and 2019FE001 (-015)]. YW was supported by the Hundred-Talent Program of Kunming Medical University (60117190439), Foundation of the Education Department of Yunnan Province (2018JS151), and the Innovation Experiment Project of Yunnan Province (202010678064) and Kunming Medical University (2020JXD014).

## Conflict of Interest

The authors declare that the research was conducted in the absence of any commercial or financial relationships that could be construed as a potential conflict of interest.

## Publisher's Note

All claims expressed in this article are solely those of the authors and do not necessarily represent those of their affiliated organizations, or those of the publisher, the editors and the reviewers. Any product that may be evaluated in this article, or claim that may be made by its manufacturer, is not guaranteed or endorsed by the publisher.
