# Supplementary material for: First Detection in West Africa of a Mutation That May Contribute to Artemisinin Resistance Plasmodium falciparum
Source: Front Genet. 2021 Oct 8;12:701750. doi: 10.3389/fgene.2021.701750 (PMC8531651; doi:10.3389/fgene.2021.701750)
Supplement: Supplementary file 1 [file Data_Sheet_1.docx]

**supplement table 1.** **Imported cases returned from regions and countries of Africa.**

| Countries | Cases | Frequency (%) |
| --- | --- | --- |
| **Western Africa** | **175** | **77.8** |
| Ghana | 164 | 73 |
| Liberia | 6 | 2.7 |
| Sierra Leone | 3 | 1.3 |
| Mali | 1 | 0.4 |
| Cote d'Ivoire | 1 | 0.4 |
| **Non-Western Africa** | **50** | **22.2** |
| Cameroon | 27 | 12 |
| Congo | 12 | 5.3 |
| Central African Republic | 6 | 2.7 |
| Gabon | 1 | 0.4 |
| Mozambique | 4 | 1.8 |
| **Total** | **225** | **100** |

**supplement table 2. Clinical characteristics of the collected cases**

| Characteristic | Total (N=225) |
| --- | --- |
|  | range |
| Age in years (Mean) | 15-64 (38.7) |
| Sex (male, female) | 217, 8 |
| Temperature (when admission at hospital) | 35.5-40.7°C |
| Hospitalization days (Mean±SD) | 3-5 (4±0.89) |
